# Does disability increase households’ health financial risk: evidence from the Uganda demographic and health survey

**DOI:** 10.1186/s41256-021-00235-x

**Published:** 2022-01-05

**Authors:** Wilfried Guets, Deepak Kumar Behera

**Affiliations:** 1 Univ Lyon, Université Lumière Lyon 2, GATE UMR 5824, 69130 Ecully, France; 2 Manipal Academy of Higher Education, Manipal, 576104 India

**Keywords:** Disability, Health financial risk, Healthcare Payment, Socio-Demographic, Household Survey, Uganda, J14, I14, J1, I32, J71, C83

## Abstract

**Background:**

In the last few years, there has been a worldwide commitment to protect the vulnerable individuals from higher financial risk through out-of-pocket (OOP) health expenditure. This study examines the influence of disability and socio-demographic factors on households’ health financial risks in Uganda.

**Methods:**

We used nationally representative cross-sectional data from the Uganda Demographic and Health Survey (UDHS) collected in 2016 by the Uganda Bureau of Statistics (UBOS) in Uganda. We measured financial risk (households’ health expenditure) by money paid for health care services. We estimated the *“probit”* model to investigate the effect of disability on health financial risk.

**Results:**

A total of 19,305 households were included in this study. Almost 32% of households paid money for health care services access, among which 32% paid through out-of-pocket. Almost 41% of household heads were affected by disability. The majority (73%) of families went to the public sector for health care services. The mean age was 45 years (SD ± 15). We find that disability is significantly associated with the household financial risk (*p* < 0.01). The private sector’s choice for health care services is likely to positively affect the financial risk compared to the public sector (*p* < 0.01). The wealthier the household was, the more money paid for health service was (*p* < 0.01).

**Conclusion:**

Our results indicated that disability and household socio-demographic characteristics were associated with health financial risk in Uganda. Identifying families with disability and experiencing difficult living conditions constitute an entry point for health authorities to enhance health coverage progress in low and middle-income countries.

## Introduction

In the last few years, there was a worldwide commitment to monitor and progress toward Universal Health Coverage (UHC in the last few years). The United Nation’s Sustainable Development Goals (SDGs) has aimed to provide universal access to healthcare for all under the health-related SDGs-3 by 2030. One of the goals is to provide financial risk protection to all vulnerable irrespective of age, gender, caste, and religion. Nevertheless, people with disabilities remain the most vulnerable group in society, but people with disabilities are not explicitly mentioned in SDG-3 to ensure healthy lives for all [1]. Few have argued that without considering people with disabilities who have more significant health needs, the achievement of universal health care may not be genuinely inclusive [2–4].

Regarding the commitment of developing countries across the globe, Uganda has not escaped this significant shift. The search for collective well-being has become a significant challenge for low-income groups, vulnerable, fragile, and disabled people. On the 25th of September 2008, Uganda’s government ratified both the convention and protocol of the United Nations Convention on the Rights of Persons with Disabilities (CRPD)^1^ [5]. Then, various plans were implemented to protect, enhance the rights, and include people with disabilities.^2^ For instance, the National Planning Authority’s (NPA) Second National Development Plan 2015/16–2019/20 (NDPII) [6, 7].

The design and implementation of government strategy for disability and inclusion in Uganda have shed light on gaps in policies and plans. Various research, such as [8], analyse the progress of disabilities policies and programs in Uganda since the last decade and explores a few critical challenges ahead. They have found that Uganda has excelled in advocating disabilities related comprehensive policies and their legal rights for healthcare services among sub-Saharan African countries. However, there seems to be an implementation gap between laws, policies, and programs. The implementation gap arises due to inadequate funding and lack of awareness leading to the exclusion of disabled people from getting any financial risk protection benefits. According to the disability statistics from the Uganda Demographic and Health Survey, on the one hand, 26% of the household population age five and above-faced difficulty at least any types of disabilities (seeing, hearing, communicating, remembering, walking, and washing) [9]. These statistics may vary according to the socio-economic and demographic characteristics of households.

On the other hand, there is less health insurance coverage (i.e. 6%) for any healthcare payment services that might constitute a significant part of out-of-pocket health expenditure and increase the financial risk burden among the vulnerable households in Uganda. Based on the latest data from [9], around 94% of men and women members of families do not have health insurance, and the percentage of women and men with insurance increased slightly from 1 to 2%, respectively, in 2011 to 6% each in 2016. This study aims to analyse the influence of disability and socio-economics and demographic characteristics on households’ healthcare services payment risk in Uganda on this above backdrop. This research includes cross-sectional and nationwide survey data in Uganda.

## Methods

### Research design

In this paper, we performed a quantitative analysis using cross-sectional survey data to investigate the effects of disability on households' health financial risk by controlling socio-economics characteristics of households heads living with disabilities in Uganda.

### Variables

We have adopted households' financial risk payment as the dependent variable in this study. Household’s health financial risk was measured by money paid to access health care services. Households’ heads were asked to answer the question*: “Do you pay any money for the services offered?”* Payment for health care includes (1) directly out-of-pocket; (2) community-based initiative/saving; (3) health insurance through employer; (4) other privately purchased commercial health insurance.^3^

The UDHS (2016) also included the DHS program disability section, a list of questions based on the Washington Group on Disability Statistics (WG) short Set referring to the International Classification of Functioning, Disability and Health (ICF) adopted by the World Health Organisation (WHO).^4^ The disability was measured following the six different functional domains: seeing, hearing, communicating, remembering or concentrating, walking or climbing steps, and washing all over or dressing (self-care).^5^ We recorded the disability variable as “1” if the respondent reported any of those.

Then, we considered determinants such as marital status, residence (rural), region, the choice of the private sector for health care, education, wealth (index), age, gender, number of children under five.

Further details regarding socio-economic and demographic characteristics are available on the Uganda DHS project.^6^

### Data sources

The Uganda Demographic and Health Survey (UDHS) was a cross-sectional, nationally representative data conducted in 2016 by the Uganda Bureau of Statistics (UBOS) between June and December 2016 [9]. The UDHS project aimed to provide recent evidence on basic demographic and health indicators (key demographic indicators such as fertility, under-five, and adult mortality; contraceptive knowledge and practice; malaria prevalence; child feeding practices; a key aspect of child and maternal health; key education indicators; extend of gender-based violence and disability.^7^ We used data based on the survey questionnaire (household), where demographic information and person characteristics were collected (age, sex, marital status, education, relationship with the household head). The UDHS also collected information on the money paid by households for health care services. In particular, the respondent provided information on the different ways they mobilised to finance health care access, out-of-pocket, community-based initiative/saving, health insurance through an employer, and other privately purchased commercial health insurance. The survey was conducted on 19,588 households. Interviews were done face-to-face across the 15 regions of the country. Further information is available on the survey website.^8^

### Data processing and analysis

Descriptive statistics were used to provide more insight into the study sample. Then, we estimated the simple and multivariate *“probit”* models to investigate characteristics and factors associated with health financial risk. We estimated the following multivariate econometric equation:

$$Financialrisk_{i} = \beta_{0} + \beta_{1} Disability_{i} + \beta_{2} X_{i} + \varepsilon_{i}$$

where $Financialrisk_{i}$ represents the dependent variable to explain. $Disability_{i}$ is a binary variable and define as *“Yes”* if the household’s head reported any form of functional limitation and *“No”* elsewhere. $X_{i}$ represents other explanatory variables. $\beta_{i}$ stands or the parameter to estimate. $\varepsilon_{i}$ is the error term.

We used the STATA SE 64 statistical software 14.2 (StataCorp, LP, College Station, TX, USA) for statistical and econometric analysis.

## Results

### Descriptive statistics

The study sample included 19,305 households in Uganda and was collected from the Uganda Demographic and Health Survey (DHS,^9^ 2016). Table 1 presents the descriptive statistics of the sample households’ socio-economic and demographic characteristics in Uganda.

**Table 1:** Descriptive statistics of socio-economic and demographic characteristics

Variables	N = 19,305 (%)	*p* value
Wealth index (%)		
Q1	4874 (25)	0.00
Q2	4848 (25)	
Q3	4831 (25)	
Q4	4752 (25)	
Marital status (%)		
Single	1257 (6)	0.00
Married	13,535 (70)	
Widowed, Divorced or Separated	4513 (24)	
Education (%)		
No education	3179 (16)	0.00
Primary	10,172 (53)	
More than secondary	5954 (31)	
Residence area (%)		
Urban	4353 (23)	0.00
Rural	14,952 (77)	
Gender (%)		
Male	13,273 (69)	0.00
Female	6032 (31)	
Region (%)		
Central	4547 (24)	0.00
Eastern	3934 (20)	
Northern	5722 (30)	
Western	5102 (26)	
Disability (%)	7979 (41)	
Pay for health care service (%)	6218 (32)	0.00
Out of pocket (%)	6123 (32)	0.00
Community-based initiative or savings (%)	26 (0.13)	0.92
Health insurance through employer (%)	95 (0.49)	0.00
Social security (%)	2 (0.01)	0.23
Private insurance (%)	14 (0.09)	0.10
The sector used for health care services (%)		
Public sector	14,081 (72.94)	0.00
Private sector	5224 (27.06)	
Age (± SD), mean	42 (15)	0.00^a^
Number of children under 5 (± SD), mean	0.97 (1.02)	0.00^a^

“*p* value” represents the test of “Chi-Squared” (Chi2) with the variable “*disability”*

^a^This result represents the “*p* value” of the Student test with the variable “*disability*”. The *p* value stands for the bivariate statistical “Chi-Squared” test for the categorical variables and the “Student” test for continuous variables. A *p* value < 5% indicated the existence of a significant relationship between the two variables tested

As indicated in Table 1, of the 19,305 households (with complete cases), almost 41% of household heads were affected by disability. Only 6% of household heads were single, 70% were married, and 23% were widowed, divorced, or separated. Almost 16% of household heads were not educated, 53% had a primary education level, and 31% more than secondary education. The majority of households were living in rural areas (77%). The majority of the heads of households were men (69%). Nearly 24% of households were in the central region, 20% in the Eastern region, 30% in the Northern region, and 26% in the Western region. Almost 33% of households paid money for health care services access, among which 32% paid through out-of-pocket, 0.13% through community-based initiative or savings, 0.5% by health insurance through an employer. The majority (73%) of households went to the public sector for health care services. The mean age was 42 years (SD ± 15). The mean of number of children under 5 years old was one per household.

### Empirical results

#### Main results

Table 2 shows the empirical results analysing factors associated with health financial risk. The multivariate econometric model (model 2) indicated that most explanatory variables were associated with health financial risk. We find that disability is significantly associated with the household financial risk (*p* < 0.01). Our results also show that the private sector choice for health care services is likely to be associated with the financial risk compared to the public sector (*p* < 0.01). Our model indicated that the wealthier the household was, the more money paid for health services was (*p* < 0.01). Married households’ heads were more likely to spend more money on health than a single (*p* < 0.1). Paying for health care services were likely to reduce with ageing (*p* < 0.01). We also find a non-linear relationship (U-shape form) between age and the financial risk in health. After 45 years [0.018/(2 * 0.0002)], we noticed that age was likely to induce a health financial risk. The regions were significantly and negatively associated with financial risk. Nevertheless, the residence area (rural), gender (female), number of children under-five were not associated with the financial risk on health. Interactions analyses indicated that the financial risk is significantly reduced with disability and for households living in the northern and western regions of the country compared to the central area.

**Table 2:** Probit model—Factors associated with the payment of health care services (per component)

	Model 1	Model 2		Model 2 bis
	Pay for health care service	Pay for health care service	Marginal effects (M.E.)	Pay for health care service with interactions
Disability	− 0.218*** (0.019)	0.076* (0.032)	0.01	0.401*** (0.112)
Private sector for health care	3.229*** (0.037)	3.136*** (0.038)	0.40	3.140*** (0.038)
Wealth—Q1	Ref	Ref		Ref
Q2	0.257*** (0.029)	0.065 (0.042)		0.063 (0.042)
Q3	0.437*** (0.028)	0.123** (0.045)	0.02	0.120** (0.045)
Q3	1.146*** (0.028)	0.341*** (0.055)	0.05	0.340*** (0.055)
Marital status—(Single)	Ref	Ref		Ref
Married	− 0.645*** (0.037)	0.165* (0.074)	0.02	0.158* (0.073)
Widowed, Divorced or Separated	− 0.730*** (0.041)	0.100 (0.081)		0.094 (0.080)
Education—(No education)	Ref	Ref		Ref
Primary	0.254*** (0.028)	− 0.026 (0.042)		− 0.030 (0.042)
More than secondary	0.706*** (0.030)	− 0.046 (0.051)		− 0.047 (0.051)
Rural	− 0.601*** (0.022)	0.027 (0.043)		0.022 (0.043)
Female	− 0.101*** (0.020)	− 0.070 (0.038)		− 0.068 (0.038)
Age	− 0.008*** (0.001)	− 0.018*** (0.005)	0.002	− 0.020*** (0.005)
Age squared	0.000*** (0.000)	0.000** (0.000)		0.000*** (0.000)
Region—(Central)	Ref	Ref		Ref
Eastern	− 0.700*** (0.028)	− 0.117** (0.045)	0.02	− 0.106 (0.059)
Northern	− 1.048*** (0.027)	− 0.266*** (0.047)	0.04	− 0.195*** (0.058)
Western	− 0.738*** (0.026)	− 0.327*** (0.042)	0.04	− 0.243*** (0.055)
Number of children under 5	− 0.099*** (0.009)	0.004 (0.014)		0.004 (0.014)
Disability * Age				− 0.005* (0.002)
Disability * Eastern				− 0.043 (0.088)
Disability * Northern				− 0.183* (0.082)
Disability * Western				− 0.200* (0.082)
Constant	− 0.374*** (0.012)	− 1.030*** (0.128)		− 1.054*** (0.130)
Number of observations	19,305	19,305	19,305	19,305

Standard errors in parentheses. Model 1 stands for univariates, whereas model 2 represents the multivariate model. M.E. represents marginal effects for the model 2

*p < 0.05; **p < 0.01; ***p < 0.001

#### Robustness checks

Further, we assumed the different components/forms of household health spending on health. We considered five types of payment sources for health care services as dependent variables: out-of-pocket spending, community-based initiative or saving, health insurance through an employer, and private insurance and examined socio-economic and demographic characteristics.

Table 3 indicated that disability significantly influenced the out-of-pocket spending (*p* < 0.01) while it likely reduces private insurance spending (*p* < 0.05). The choice for the private sector for health care significantly affected the health expenditure; this effect was significant for out-of-pocket (*p* < 0.01), community-based initiative or savings (*p* < 0.01), health insurance through an employer (*p* < 0.01), and private insurance (*p* < 0.01). Households in the highest wealth quintile were more likely to spend more money on health services (*p* < 0.01). Being married have a positive influence on out-of-pocket spending (*p* < 0.05). The age was negatively associated with out-of-pocket (*p* < 0.01). Conversely, age is significantly associated with health insurance spending through an employer (*p* < 0.1). For health care access, families in the Western region of the country, in particular, were more likely to pay less in terms of out-of-pocket (*p* < 0.01) but contribute more in terms of community-based initiative or savings (*p* < 0.01).

**Table 3:** Probit model—Factors associated with the payment of health care services across source healthcare spending

	(1) Out of pocket	(2) Community-based initiative or savings	(3) Health insurance through an employer	(4) Private insurance
Disability	0.092** (0.031)	0.039 (0.150)	− 0.248 (0.128)	− 0.423** (0.154)
Private sector for health care	2.970*** (0.034)	0.959*** (0.166)	0.721*** (0.115)	1.038*** (0.295)
Q1	Ref	Ref	Ref	Ref
Q2	0.076 (0.041)	− 0.147 (0.287)	− 0.095 (0.163)	− 0.274 (0.244)
Q3	0.151*** (0.043)	0.264 (0.253)	− 0.344* (0.168)	0.000^a^ –
Q3	0.311*** (0.054)	0.075 (0.271)	0.000^a^ –	0.000^a^ –
Single	Ref	Ref	Ref	Ref
Married	0.216** (0.071)	− 0.234 (0.275)	− 0.080 (0.146)	− 0.339 (0.270)
Widowed, divorced or separated	0.176* (0.077)	− 0.362 (0.284)	− 0.341 (0.183)	− 0.646* (0.311)
No education	Ref	Ref	Ref	Ref
Primary	− 0.019 (0.041)	0.134 (0.256)	− 0.582*** (0.133)	0.198 (0.374)
More than secondary	− 0.090 (0.050)	0.230 (0.292)	0.000^a^ –	0.369 (0.327)
Rural	0.068 (0.042)	0.073 (0.170)	− 0.406*** (0.101)	0.116 (0.147)
Female	− 0.073* (0.036)	− 0.074 (0.188)	0.088 (0.103)	0.478** (0.161)
Age	− 0.019*** (0.005)	0.008 (0.027)	0.050* (0.024)	0.025 (0.028)
Age squared	0.000*** (0.000)	− 0.000 (0.000)	− 0.001 (0.000)	− 0.000 (0.000)
Central	Ref	Ref	Ref	Ref
Eastern	− 0.076 (0.044)	0.000^a^ –	− 0.475* (0.195)	0.000^a^ –
Northern	− 0.224*** (0.046)	0.521 (0.349)	− 0.163 (0.156)	0.000^a^ –
Western	− 0.299*** (0.041)	1.206*** (0.281)	− 0.113 (0.127)	0.290* (0.131)
Number of children under 5	0.010 (0.014)	0.039 (0.059)	− 0.194* (0.081)	0.082 (0.102)
Constant	− 1.117*** (0.124)	− 4.671*** (0.563)	− 3.171*** (0.468)	− 4.753*** (0.592)
Number of observations	19,305	15,371	12,729	5882

Standard errors in parentheses. The different components are: (1) Directly out-of-pocket; (2) community-based initiative/saving; (3) health insurance through an employer; (4) other privately purchased commercial health insurance

*p < 0.05; **p < 0.01; ***p < 0.001

^a^Not estimated due to the weakness of sample and variable contains many missing values. Variables “*Community-based initiative/saving”, “health insurance through an employer”, “other privately purchased commercial health insurance”* contain missing data

## Discussion

This study reports finding based on nationally representative data from the Uganda Demographic and Health Survey (UDHS) on the various healthcare services payments utilisation by households associated with disability. To reduce financial risk due to healthcare services, these vulnerable groups should be prioritised to achieve UHC [1, 10]. However, limited research has been conducted to investigate the impact of disability and other socio-demographic factors on households’ financial risk due to healthcare payment services in sub-Saharan African countries as Uganda, where 26% of the households fall into disabilities [8, 11, 12]. The majority of studies analysed the burden of chronic illness and multimorbidity on out-of-pocket health expenditure and measured catastrophic [3, 13, 14]. Very few tried to link disability and poverty relationships by measuring impoverishment in the same vein [2, 15]. Therefore, we examined the impact of disabilities and socio-demographic characteristics on the households’ financial risk on healthcare payment across payment sources: out-of-pocket spending, community-based initiative or saving, health insurance through an employer, and private insurance.

In this study, we found that around 41% of household heads were affected by disability, and the majority of them have lived in rural areas, and men centric disabilities are more. Almost 32% of households paid for healthcare services through their own out-of-pocket. A very negligible number of households were covered with health insurance (i.e. community-based or private health insurance). Our results concord with earlier studies finding that disability households usually face catastrophic medical expenses due to higher out-of-pocket payment services and low insurance payment coverage [3, 13, 15]. Specifically, wealthier households and married individuals are likely to spend more money on healthcare services than lower-income households and single-headed households. This relationship is evident because positive income elasticity of health spending might treat healthcare services as a normal good than the counterpart [16, 17]. We find no financial risk associated with those disability households residing in rural areas, female-headed households, and children under 5 years. This is a fascinating result and contrast view from the earlier literature. Some studies found that households living in a rural area and female participants were highly prone to financial risk due to higher out-of-pocket health payments [14, 18, 19].

We have also found a close association between the choice of private healthcare payments and age categories. These findings shed light on the non-linear impact of age on out-of-pocket health spending. Younger age reduced out-of-pocket and opted for other payment sources such as employee health insurance. In comparison, in older age (i.e. age squared), health services’ payment is derived from out-of-pocket. These results are similar to earlier finding that ageing is one of the pertinent factors for higher out-of-pocket payment, and it remains a debatable issue in health economics.

Conversely, few studies find that ageing has no longer a problem for households’ catastrophic health payments in the Asia–pacific countries. They presumed that it might be a problem in the long run when these countries would move any demographic or epidemiological transition [17, 20]. The results also highlighted a huge variation in the healthcare payment system across geographic regions of Uganda. For instance, the Western region has more access to community-based insurance and private insurance, which depends on the household’s pocket expenditure.

The overall discussion concludes that out-of-pocket payment have a positive and significant effect on the financial risk of disabled households. A significantly less proportion of disabilities households reduce their financial risk through other payment modes—private insurance that only for higher wealth quintile households. Additionally, other socio-demographic factors impact household financial risk protection by using different healthcare payment services, including ageing, socio-economic and development of regions in Uganda, and lower dependency households. Our results are following other studies investigating the association between disabilities and catastrophic health payment [3, 13, 21].

Given that, our study used nationwide data sets, there is no denying that it has several limitations. Our work relies on cross-sectional data, and therefore, morbidity due to disabilities and healthcare payment, and other variables or data were not easy to capture in this paper. Furthermore, our finding is based on self-reported healthcare payment and disabilities, which might cause a lag effect of the households’ financial crisis or any recall bias related to healthcare payment. Some cautions should be taken regarding the dependent variable (private insurance) because many respondents did not/refused to answer related questions.

## Conclusion

This study examined the influence of disability and socio-demographic factors on households’ health financial risks in Uganda. The findings show that disability and household socio-demographic characteristics were associated with health financial risk in Uganda. Identifying families with disabilities and experiencing difficult living conditions constitute an entry point for health authorities to enhance health coverage progress in low and middle-income countries. Despite a few limitations, our study fills a significant knowledge gap. It relied on cross-sectional and nationally representative data sets to estimate the effect of disability on households' healthcare services payments regarding different payment sources. These results have implications for health policies. One should design effective disability-health related policies in sub-Saharan African countries. Priorities should target fragile, vulnerable populations and groups with a high risk of financial impoverishment.

## Data Availability

The datasets supporting this research’s finding are available in the Demographic and Health Surveys (DHS) Program repository. https://dhsprogram.com/pubs/pdf/FR333/FR333.pdf; https://www.dhsprogram.com/

## Abbreviations

UBOS: Uganda bureau of statistics
UDHS: Uganda demographic and health survey
UHC: Universal health coverage
OOP: Out-of-pocket

## Footnotes

1 The Uganda Persons with Disability Act 2006 considered disability as a “substantial functional limitation of daily life activities caused by physical, mental or sensory impairment and environmental barriers resulting in limited participation.”.

2 The National Planning Authority’s (NPA) Second National Development Plan 2015/16–2019/20 (NDPII) and the MoGLSD’s Social Development Sector Plan 2015/16–2019/20 (SDSP) illustrate this.

3 It is important to note the number of families having access to health insurance through employer; social security; and other privately purchased commercial health insurance were quite low in Uganda.

4 https://www.who.int/standards/classifications/international-classification-of-functioning-disability-and-health/who-disability-assessment-schedule.

5 This approach provides basic essential information on disability comparable to those collected worldwide via Washington Group (WG) disability tools. Based on the household questionnaire, respondents were aged 15 or above.

6 https://dhsprogram.com/data/dataset/Uganda_Standard-DHS_2016.cfm?flag=0.

7 https://dhsprogram.com/pubs/pdf/FR333/FR333.pdf.

8 https://dhsprogram.com/data/dataset/Uganda_Standard-DHS_2016.cfm?flag=0.

9 The 2016 Uganda Demographic and Health Survey (2016 DHS) was implemented by Uganda Bureau of Statistics, Government of Uganda. This survey provides information on population health and nutrition programme in every 5 years.
